# Supplementary material for: In-Silico Investigation of Effects of Single-Nucleotide Polymorphisms in PCOS-Associated CYP11A1 Gene on Mutated Proteins
Source: Genes (Basel). 2022 Jul 12;13(7):1231. doi: 10.3390/genes13071231 (PMC9317558; doi:10.3390/genes13071231)
Supplement: Supplementary file 1 [file genes-13-01231-s001.zip › genes-1779223-supplementary.pdf]

Case 1

M L A K G L P P R S V L V K G C Q T F L S A P R E G L G R L R V P T G E G A G I
S T R S P R P F N E I P S P G D N G W L N L Y H F W R E T G T H K V H L H H V Q
N F Q K M A R F T G R S S A T W S R F M S S T L K M W P F S L S P R A P T Q N D
S S S R P G S P I T S I T R D P

Case 2

M L A K G L S P R S V L V K G C Q T F M S A P R E G L G R L R V P T G E G A G T
F T R S P R P F N E I P S P G D N G W V K L Y H F W R E T G T H K V H L H H I Q
N L Q K Y G P I Y R K K L S N V E S V Y V I D P E N V A L L F K S E G P N P E R
F L I P P W V A Y H Q Y Y Q R P I G V L L K K T A A W R K D R V A L N Q E V M A
P G A T K N F L P L L D A V S R D F V S V P H R R I K K A G S G N Y S G D I S D
D L F R F A F K S I T N V I F G E R Q G M L E E V V N P E A Q R F I D A I Y Q M F
H T S V P M L N L P P D L F R L F R T K T W K D H V A A W D V I F S K A D I Y T
Q N F Y W E L R Q K G S V H H D Y R G I L Y R L L G D S K M S F E D I K A N V T
E M L A G G V D T T S M T L Q W H L Y E M A R N L K V Q D M L R A E V L A A R H
Q A Q G D M A T M L Q L V P L L K A S I K E T L R L H P I S V T L Q R Y L V N D
L V L R D Y M I P A K T L V Q V A I Y A L G R E P T F F F D P E N F D P T R W L
S K D K N I T Y F R N L G F G W G V R Q C L G R R I A E L E M T I F L I N M L E
N F R V E I Q H L S D V G T T F N L I L M P E K P I S F T F W P F N Q E A T Q Q

Case 3

M L A K G L P P R S V L V K G C Q T F L S A P R E G L G R L R V P T G E G A G I
S T R S P R P F N E I P S P G D N G W L N L Y H F W R E T G T H K V H L H H V Q
N F Q K Y G P I Y R E K L G N V E S V Y V I D P E D V A L L F K S E G P N P E

Case 4

M L A K G L P P R S V L V K G C Q T F L S A P R E G L G R L R V P T G E G A G I
S T R S P R P F N E I P S P G D N G W L N L Y H F W R E T G T H K V H L H H V Q
N F Q K Y G P I Y R E K L G N V E S V Y V S T L K M W P F S L S P R A Q P R T I
P H P A L G R L S P V L P E T H R S P V E E V G S L E E R P G G P E P G G D G S
R G H Q E L F A P V G C S V S G L R Q C P A Q A H Q E G G L R K L L G G H Q

Case 5

M L A K G L P P R S V L V K G C Q T F L S A P R E G L G R L R V P T G E G A G I
S T R S P R P F N E I P S P G D N G W L N L Y H F W R E T G T H K V H L H H V Q
N F Q K Y G P I Y R E K L G N V E S V Y V I D P E D V A L L F K S E G P N P E R
F L I P P W V A Y P P V L P E T H R S P V E E V G S L E E R P G G P E P G G D G
S R G H Q E L F A P V G C S V S G L R Q C P A Q A H Q E G G L R K L L G G H Q

Case 6

M L A K G L P P R S V L V K G C Q T F L S A P R E G L G R L R V P T G E G A G I
S T R S P R P F N E I P S P G D N G W L N L Y H F W R E T G T H K V H L H H V Q
N F Q K Y G P I Y R E K L G N V E S V Y V I D P E D V A L L F K S E G P N P E R
F L I P P W V A Y H Q Y Y Q R P I G V L L K K

Case 7

M L A K G L P P R S V L V K G C Q T F L S A P R E G L G R L R V P T G E G A G I
S T R S P R P F N E I P S P G D N G W L N L Y H F W R E T G T H K V H L H H V Q
N F Q K Y G P I Y R E K L G N V E S V Y V I D P E D V A L L F K S E G P N P E R
F L I P P W V A Y H Q Y Y Q R P I G V L L K K S A A W K K D R V A L N Q E V M A
P E A T K N F L P L L D A C L G T S S V S C T G A S R R R A P E I T R G T S
V Met T C S A L P L S P S L T S F L G S A R G C W R K

Case 8

M L A K G L P P R S V L V K G C Q T F L S A P R E G L G R L R V P T G E G A G I
S T R S P R P F N E I P S P G D N G W L N L Y H F W R E T G T H K V H L H H V Q
N F Q K Y G P I Y R E K L G N V E S V Y V I D P E D V A L L F K S E G P N P E R
F L I P P W V A Y H Q Y Y Q R P I G V L L K K S A A W K K D R V A L N Q E V M A
P E A T K N F L P R W M Q C L G T S S V S C T G A S R R R A P E I T R G T S
V Met T C S A L P L S P S L T S F L G S A R G C W R K

Case 9

M L A K G L P P R S V L V K G C Q T F L S A P R E G L G R L R V P T G E G A G I
S T R S P R P F N E I P S P G D N G W L N L Y H F W R E T G T H K V H L H H V Q
N F Q K Y G P I Y R E K L G N V E S V Y V I D P E D V A L L F K S E G P N P E R
F L I P P W V A Y H Q Y Y Q R P I G V L L K K S A A W K K D R V A L N Q E V M A
P E A T K N F L P L L D A V S R D F V S V L H R R I K K A G S G N Y S G D I S D
D L F R F A F E S I T N V I F G E R Q G M L E E V V N P E V Q R F I D A V Y Q M F
H T S V P M P N L P P D L F R L L R T K A W K D H V S V W D V I F S K A D I Y T
Q N S Y R E L R Q K G S V H H D S R G I L Y R L L G D S K M S F E D I K A N V T
E Met V E G G V G M M S T T L Q W H L F K M A R N L K V Q D M L R A E V L A A R
H Q A Q G D M A T M L R L V P L L K A S I K E T L R L H P I S V T L Q R Y L V N
D L V L R D Y M I P A K T L V Q V A I Y A L G R E P T F F F D P E N F D P T R W
L S K D K N I T Y F R N L G F G W G V R Q C L G R R I A E L E M T I F L I N Met L
E N F R V E I Q H L S D V G T T F N L I L M P E K P I S F T F W P F N Q E A T Q
Q

Case 10

M L A K G L P P R S V L V K G C Q T F L S A P R E G L G R L R V P T G E G A G I
S T R S P R P F N E I P S P G D N G W L N L Y H F W R E T G T H K V H L H H V Q
N F Q K Y G P I Y R E K L G N V E S V Y V I D P E D V A L L F K S E G P N P E R
F L I P P W V A Y H Q Y Y Q R P I G V L L K K S A A W K K D R V A L N Q E V M A

P E A T K N F L P L L D A V S R D F V S V L H R R I K K A G S G N Y S G D I S D
D L F R F A F E S I T N V I F G E R Q G M L E E V V N P E A Q

Case 11

M L A K G L P P R S V L V K G C Q T F L S A P R E G L G R L R V P T G E G A G I
S T R S P R P F N E I P S P G D N G W L N L Y H F W R E T G T H K V H L H H V Q
N F Q K Y G P I Y R E K L G N V E S V Y V I D P E D V A L L F K S E G P N P E R
F L I P P W V A Y H Q Y Y Q R P I G V L L K K S A A W K K D R V A L N Q E V M A
P E A T K N F L P L L D A V S R D F V S V L H R R I K K A G S G N Y S G D I S D
D L F R F A F E S I T N V I F G E R Q G M L E E V V N P E A Q R F I D A I Y Q M F
H T S V P M L N L P P D L F R L F R T K T W K D H V A A W D V I F S K A D I Y T

Case 12

M L A K G L P P R S V L V K G C Q T F L S A P R E G L G R L R V P T G E G A G I
S T R S P R P F N E I P S P G D N G W L N L Y H F W R E T G T H K V H L H H V Q
N F Q K Y G P I Y R E K L G N V E S V Y V I D P E D V A L L F K S E G P N P E R
F L I P P W V A Y H Q Y Y Q R P I G V L L K K S A A W K K D R V A L N Q E V M A
P E A T K N F L P L L D A V S R D F V S V L H R R I K K A G S G N Y S G D I S D
D L F R F A F E S I T N V I F G E R Q G M L E E V V N P E A Q R F I D A I Y Q M F
H T S V P M L N L P P D L F R L F R T K T W K D H V A A W D V I F S K A D Y T P
R T S T G N

Case 13

M L A K G L P P R S V L V K G C Q T F L S A P R E G L G R L R V P T G E G A G I
S T R S P R P F N E I P S P G D N G W L N L Y H F W R E T G T H K V H L H H V Q
N F Q K Y G P I Y R E K L G N V E S V Y V I D P E D V A L L F K S E G P N P E R
F L I P P W V A Y H Q Y Y Q R P I G V L L K K S A A W K K D R V A L N Q E V M A
P E A T K N F L P L L D A V S R D F V S V L H R R I K K A G S G N Y S G D I S D

D L F R F A F E S I T N V I F G E R Q G M L E E V V N P E A Q R F I D A I Y Q M F
H T S V P M L N L P P D L F R L F R T K T W K D H V A A W D V I F S K A D I Y T
Q N F Y W E L R Q K G S V H H D Y R G I L Y R L L G D S K M S F R T S R P T S Q
R C W Q E G G H D V H D P A V A L V

Case 14

M L A K G L P P R S V L V K G C Q T F L S A P R E G L G R L R V P T G E G A G I
S T R S P R P F N E I P S P G D N G W L N L Y H F W R E T G T H K V H L H H V Q
N F Q K Y G P I Y R E K L G N V E S V Y V I D P E D V A L L F K S E G P N P E R
F L I P P W V A Y H Q Y Y Q R P I G V L L K K S A A W K K D R V A L N Q E V M A
P E A T K N F L P L L D A V S R D F V S V L H R R I K K A G S G N Y S G D I S D
D L F R F A F E S I T N V I F G E R Q G M L E E V V N P E A Q R F I D A I Y Q M F
H T S V P M L N L P P D L F R L F R T K T W K D H V A A W D V I F S K A D I Y T
Q N F Y W E L R Q K G S V H H D Y R G I L Y R L L G D S K M S F E D I K A N V T
E M L A G G V D T T S M T L Q W H L Y E M A R N L K V Q D M L R A E V L A A R H
Q A Q G D M A T M L Q L V P L L K A S I K E T L R L Y P I P V T L K R Y V L N D
L F I R H Y M T P T K T L V E V A I C A L G R E P T F F F D P E N F D S T R W L
S K D K N I T Y F R N L G F G W G V R Q C P G R R I T E L E V T V F L I N L L E
N F R I E I Q H L S D E G T T F N L I L T L E K P I S F T F W S L I Q E A T Q Q

Case 15

M L A K G L P P R S V L V K G C Q T F L S A P R E G L G R L R V P T G E G A G I
S T R S P R P F N E I P S P G D N G W L N L Y H F W R E T G T H K V H L H H V Q
N F Q K Y G P I Y R E K L G N V E S V Y V I D P E D V A L L F K S E G P N P E R
F L I P P W V A Y H Q Y Y Q R P I G V L L K K S A A W K K D R V A L N Q E V M A
P E A T K N F L P L L D A V S R D F V S V L H R R I K K A G S G N Y S G D I S D
D L F R F A F E S I T N V I F G E R Q G M L E E V V N P E A Q R F I D A I Y Q M F
H T S V P M L N L P P D L F R L F R T K T W K D H V A A W D V I F S K A D I Y T

Q N F Y W E L R Q K G S V H H D Y R G I L Y R L L G D S K M S F E D I K A N V T
E M L A G G V D T T S M T L Q W H L Y E M A R N L K V Q D M L R A E V L A A R H
Q A Q G D M A T M L Q L V P L L K A S I K E T L R L H P I S V T L Q R Y L V N D
L V L

Case 16

Met L A K G L P P R S V L V K G C Q T F L S A P R E G L G R L R V P T G E G A G
I S T R S P R P F N E I P S P G D N G W L N L Y H F W R E T G T H K V H L H H V
Q N F Q K Y G P I Y R E K L G N V E S V Y V I D P E D V A L L F K S E G P N P E
R F L I P P W V A Y H Q Y Y Q R P I G V L L K K S A A W K K D R V A L N Q E VMet A
P E A T K N F L P L L D A V S R D F V S V L H R R I K K A G S G N Y S G D I S D
D L F R F A F E S I T N V I F G E R Q G Met L E E V V N P E A Q R F I D A I Y
Q Met F H T S V P Met L N L P P D L F R L F R T K T W K D H V A A W D V I F S K
A D I Y T Q N F Y W E L R Q K G S V H H D Y R G I L Y R L L G D S K Met S F E D
I K A N V T E Met L A G G V D T T S Met T L Q W H L Y E Met A R N L K V Q D Met L
R A E V L A A R H Q A Q G D Met A T Met L Q L V P L L K A S I K E T L R L H P I
S V T L Q R Y L V N D L V L R D Y Met I P A K T L V Q V A I Y A L G

Case 17

Met L A K G L P P R S V L V K G C Q T F L S A P R E G L G R L R V P T G E G A G
I S T R S P R P F N E I P S P G D N G W L N L Y H F W R E T G T H K V H L H H V
Q N F Q K Y G P I Y R E K L G N V E S V Y V I D P E D V A L L F K S E G P N P E
R F L I P P W V A Y H Q Y Y Q R P I G V L L K K S A A W K K D R V A L N Q E VMet A
P E A T K N F L P L L D A V S R D F V S V L H R R I K K A G S G N Y S G D I S D
D L F R F A F E S I T N V I F G E R Q G Met L E E V V N P E A Q R F I D A I Y
Q Met F H T S V P Met L N L P P D L F R L F R T K T W K D H V A A W D V I F S K
A D I Y T Q N F Y W E L R Q K G S V H H D Y R G I L Y R L L G D S K Met S F E D
I K A N V T E Met L A G G V D T T S Met T L Q W H L Y E Met A R N L K V Q D Met L

R A E V L A A R H Q A Q G D Met A T Met L Q L V P L L K A S I K E T L R L H P I
S V T L Q R Y L V N D L V L R D Y Met I P A K T L V Q V A I Y A L G R E P T F F
F D P E N F D P T Stop

Case 18

Met L A K G L P P R S V L V K G C Q T F L S A P R E G L G R L R V P T G E G A G
I S T R S P R P F N E I P S P G D N G W L N L Y H F W R E T G T H K V H L H H V
Q N F Q K Y G P I Y R E K L G N V E S V Y V I D P E D V A L L F K S E G P N P E
R F L I P P W V A Y H Q Y Y Q R P I G V L L K K S A A W K K D R V A L N Q E VMet A
P E A T K N F L P L L D A V S R D F V S V L H R R I K K A G S G N Y S G D I S D
D L F R F A F E S I T N V I F G E R Q G Met L E E V V N P E A Q R F I D A I Y
Q Met F H T S V P Met L N L P P D L F R L F R T K T W K D H V A A W D V I F S K
A D I Y T Q N F Y W E L R Q K G S V H H D Y R G I L Y R L L G D S K Met S F E D
I K A N V T E Met L A G G V D T T S Met T L Q W H L Y E Met A R N L K V Q D Met L
R A E V L A A R H Q A Q G D Met A T Met L Q L V P L L K A S I K E T L R L H P I
S V T L Q R Y L V N D L V L R D Y Met I P A K T L V Q V A I Y A L G R E P T F F
F D P E N F D P T R W L S K D K N I T Y F R N L G F G W G V R Q C L G R R I A E
L E Met T I F L I N Met L E N F R V E I Q H L S D V G T T F N L I L Met P E K P
I S F T F W P F N Q E A T Q Stop

Case 19

Met L A K G L P P R S V L V K G C Q T F L S A P R E G L G R L R V P T G E G A G I
S T R S P R P F N E I P S P G D N G W L N L Y H F W R E T G T H K V H L H H V Q
N F Q K Y G P I Y R E K L G N V E S V Y V I D P E D V A L L F K S E G P N P E R
F L I P P W V A Y H Q Y Y Q R P I G V L L K K S A A W K K D R V A L N Q E VMet A
P E A T K N F L P L L D A V S R D F V S V L H R R I K K A G S G N Y S G D I S D
D L F R F A F E S I T N V I F G E R Q G Met L E E V V N P E A Q R F I D A I Y
Q Met F H T S V P Met L N L P P D L F R L F R T K T W K D H V A A W D V I F S K A

D I Y T Q N F Y W E L R Q K G S V H H D Y R G I L Y R L L G D S K Met S F E D I K
A N V T E Met L A G G V D T T S Met T L Q W H L Y E Met A R N L K V Q D Met L R A E
V L A A R H Q A Q G D Met A T Met L Q L V P L L K A S I K E T L R L H P I S V T L
Q R Y L V N D L V L R D Y Met I P A K T L V Q V A I Y A L G R E P T F F F D P E N
F D P T R W L S K D K N I T Y F R N L G F G W G V R Q C L G R R I A E L E Met T I
F L I N Met L E N F R V E I Q H L S D V G T T F N L I L Met P E K P I S F T F W P
F N Q E A T Q Q R

Case 20

Met L A K G L P P R S V L V K G C Q T F L S A P R E G L G R L R V P T G E G A G
I S T R S P R P F N E I P S P G D N G W L N L Y H F W R E T G T H K V H L H H V
Q N F Q K Y G P I Y R E K L G N V E S V Y V I D P E D V A L L F K S E G P N P E
R F L I P P W V A Y H Q Y Y Q R P I G V L L K K S A A W K K D R V A L N Q E VMet A
P E A T K N F L P L L D A V S R D F V S V L H R R I K K A G S G N Y S G D I S D
D L F R F A F E S I T N V I F G E R Q G Met L E E V V N P E A Q R F I D A I Y
Q Met F H T S V P Met L N L P P D L F R L F R T K T W K D H V A A W D V I F S K
A D I Y T Q N F Y W E L R Q K G S V H H D Y R G I L Y R L L G D S K Met S F E D
I K A N V T E Met L A G G V D T T S Met T L Q W H L Y E Met A R N L K V Q D Met L
R A E V L A A R H Q A Q G D Met A T Met L Q L V P L L K A S I K E T L R L H P I
S V T L Q R Y L V N D L V L R D Y Met I P A K T L V Q V A I Y A L G R E P T F F
F D P E N F D P T R W L S K D K N I T Y F R N L G F G W G V R Q C L G R R I A E
L E Met T I F L I N Met L E N F R V E I Stop

Case 21

Met L A K G L P P R S V L V K G C Q T F L S A P R E G L G R L R V P T G E G A G
I S T R S P R P F N E I P S P G D N G W L N L Y H F W R E T G T H K V H L H H V
Q N F Q K Y G P I Y R E K L G N V E S V Y V I D P E D V A L L F K S E G P N P E

R F L I P P W V A Y H Q Y Y Q R P I G V L L K K S A A W K K D R V A L N Q E VMet A
P E A T K N F L P L L D A V S R D F V S V L H R R I K K A G S G N Y S G D I S D
D L F R F A F E S I T N V I F G E R Q G Met L E E V V N P E A Q R F I D A I Y
Q Met F H T S V P Met L N L P P D L F R L F R T K T W K D H V A A W D V I F S K
A D I Y T Q N F Y W E L R Q K G S V H H D Y R G I L Y R L L G D S K Met S F E D
I K A N V T E Met L A G G V D T T S Met T L Q W H L Y E Met A R N L K V Q D Met L
R A E V L A A R H Q A Q G D Met A T Met L Q L V P L L K A S I K E T L R L H P I
S V T L Q R Y L V N D L V L R D Y Met I P A K T L V Q V A I Y A L G R E P T F F
F D P E N F D P T R W L S K D K N I T Y F R N L G F G W G V R Q C L G R R I A E
L E Met T I F L I N Met L E N F R V E I Q H L S D V G T T F N L I L Met P E K P
I S F T F W P F N Stop

Case 22

Met L A K G L P P R S V L V K G C Q T F L S A P R E G L G R L R V P T G E G A G
I S T R S P R P F N E I P S P G D N G W L N L Y H F W R E T G T H K V H L H H V
Q N F Q K Y G P I Y R E K L G N V E S V Y V I D P E D V A L L F K S E G P N P E
R F L I P P W V A Y H Q Y Y Q R P I G V L L K K S A A W K K D R V A L N Q E VMet A
P E A T K N F L P L L D A V S R D F V S V L H R R I K K A G S G N Y S G D I S D
D L F R F A F E S I T N V I F G E R Q G Met L E E V V N P E A Q R F I D A I Y
Q Met F H T S V P Met L N L P P D L F R L F R T K T W K D H V A A W D V I F S K
A D I Y T Q N F Y W E L R Q K G S V H H D Y R G I L Y R L L G D S K Met S F E D
I K A N V T E Met L A G G V D T T S Met T L Q W H L Y E Met A R N L K V Q D Met L
R A E V L A A R H Q A Q G D Met A T Met L Q L V P L L K A S I K E T L R L H P I
S V T L Q R Y L V N D L V L R D Y Met I P A K T L V Q V A I Y A L G R E P T F F
F D P E N F D P T R W L S K D K N I T Y F R N L G F G W G V R Q C L G R R I A E
L E Met T I F L I N Met L E N F R V E I Q H L S D V G T T F N L I L Met P E K P
I S F T F W P F N Q

Figure S1: Protein sequences of mutated proteins retrieved through Expasy tool for cases 1-22, containing variations documented in present study

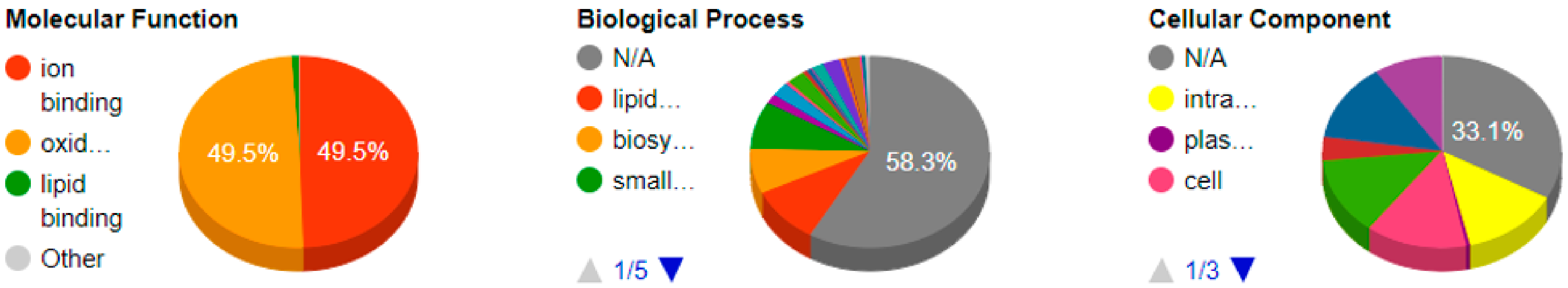

Case 17

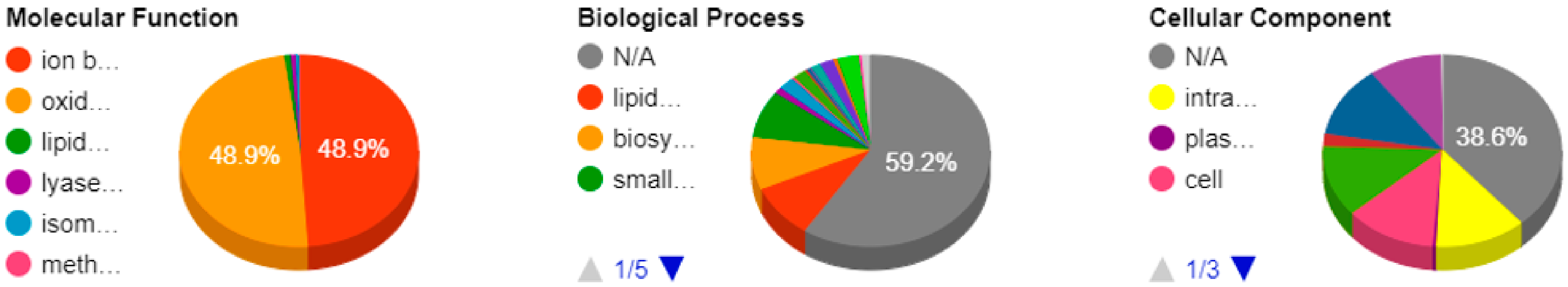

Case 18

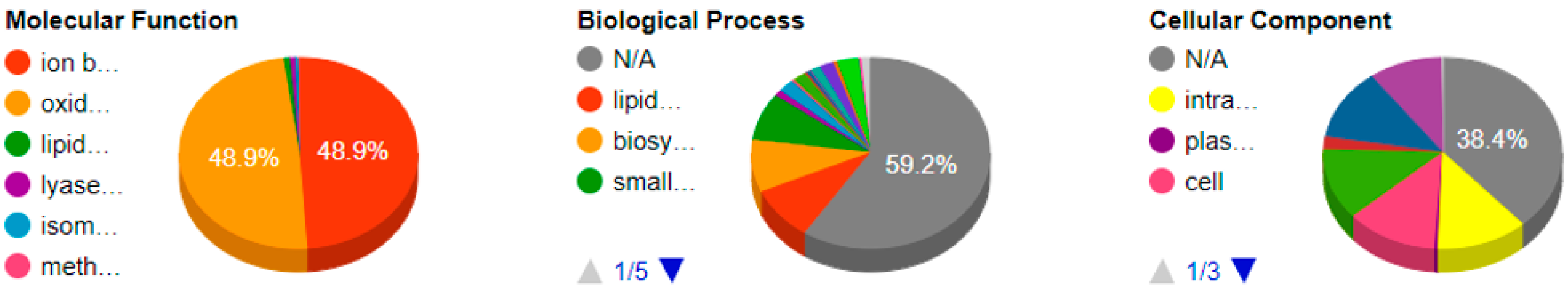

Case 19

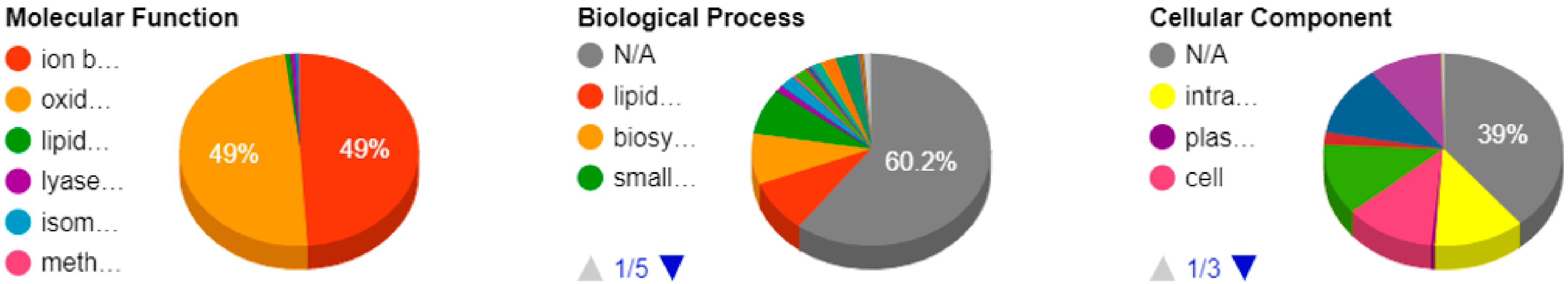

Case 20

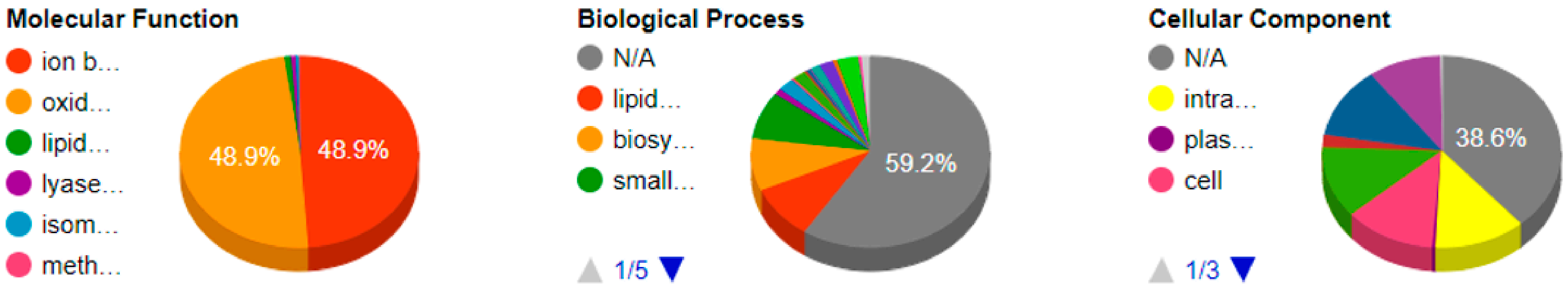

Case 21

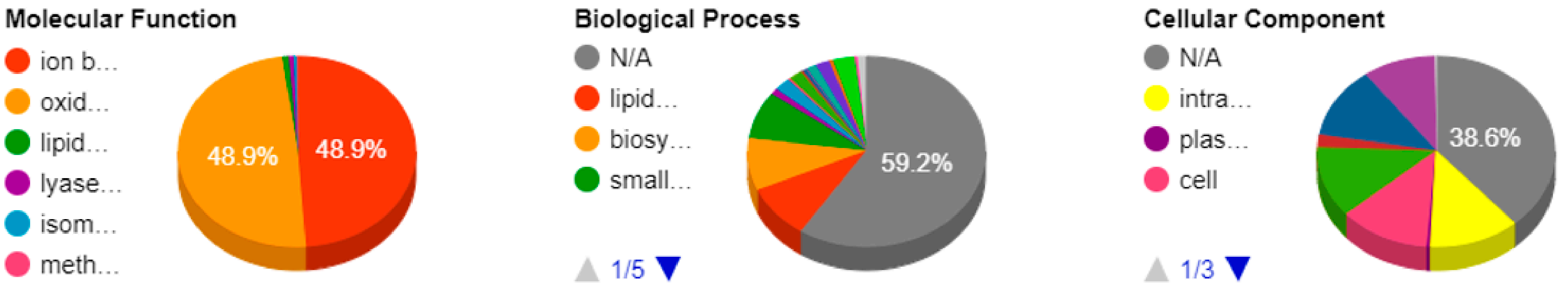

Case 22

Figure S2: Effect of SNPs documented in cases 17-22 on the ontology of mutated proteins

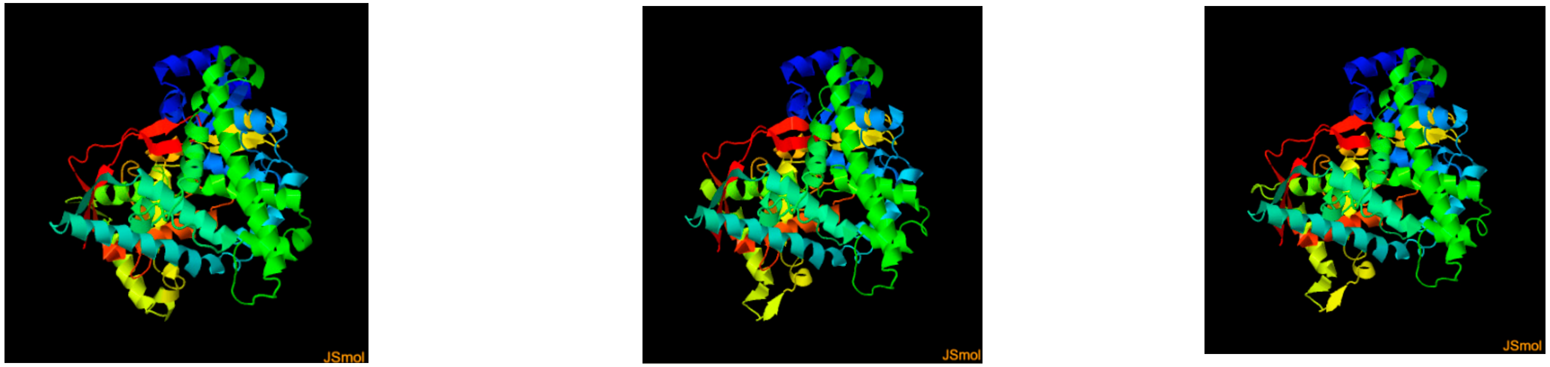

Case 2 **Case 9** **Case 14**

Figure S3: Effect of SNPs documented in cases 2, 9 and 14 on 3D structure of mutated proteins

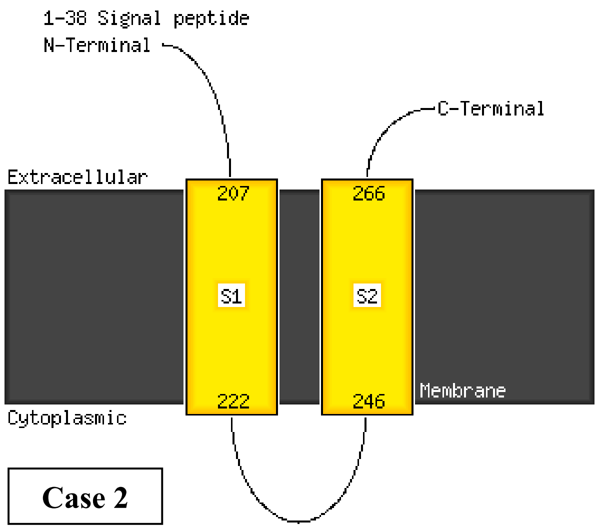

Case 2

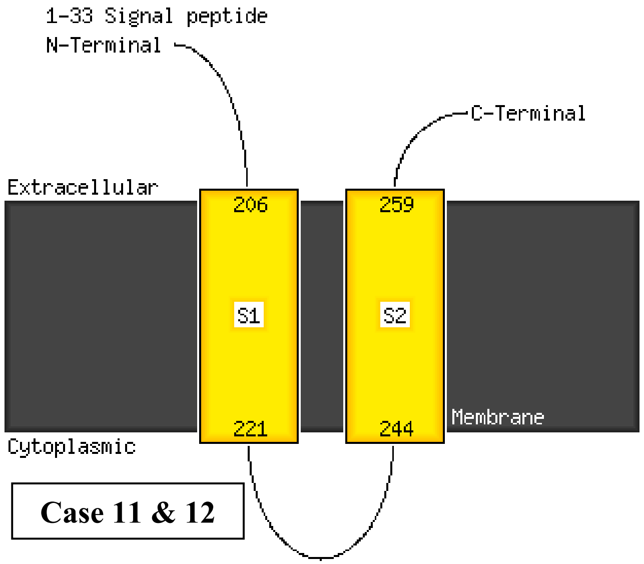

Case 11 & 12

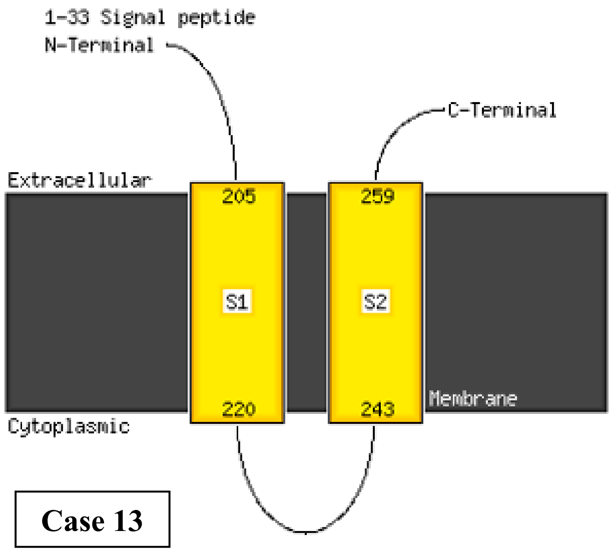

Case 13

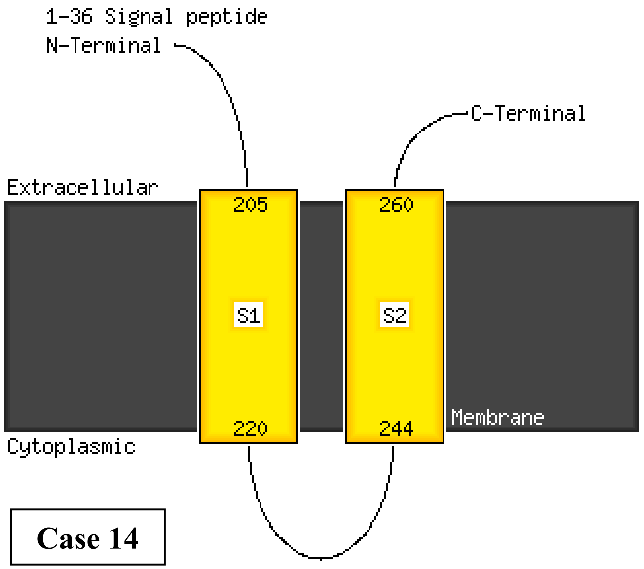

Case 14

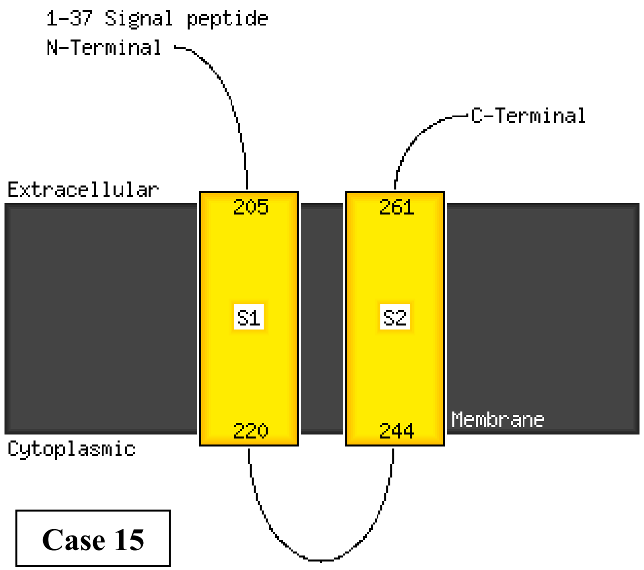

Case 15

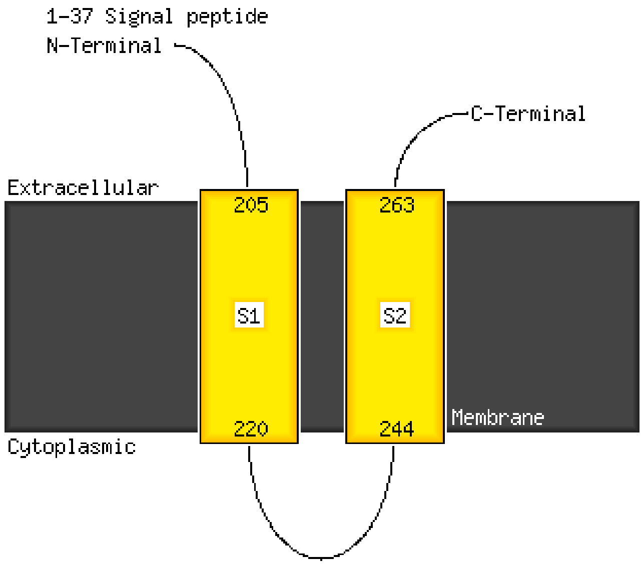

Case 16, 17 & 22

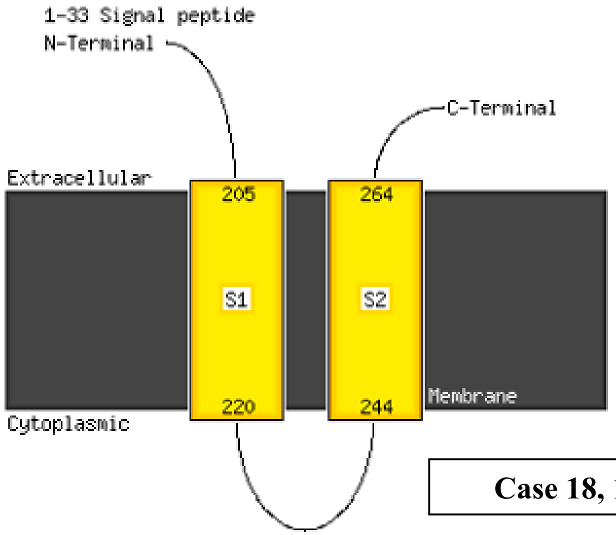

Case 18, 19, 20 & 21

Figure S4: Effect of SNPs documented in cases 2 and 11-22 on the topology of mutated proteins

Table S1: Variations in 2D structure of mutated proteins induced by SNPs documented in present study

Case ID	Disorder (%)	Alpha helix (%)	Beta strand (%)
**Normal**	22	50	9
**Case 1**	44	38	6
**Case 2**	23	51	9
**Case 3**	41	50	8
**Case 4**	37	58	5
**Case 5**	37	50	6
**Case 6**	34	43	7
**Case 7**	25	62	4
**Case 8**	24	63	4
**Case 9**	22	50	9
**Case 10**	24	58	4
**Case 11**	23	62	3
**Case 12**	22	62	3
**Case 13**	24	63	3

**Case 14**	22	51	9
**Case 15**	25	62	4
**Case 16**	22	57	5
**Case 17**	21	57	6
**Case 18**	21	51	9
**Case 19**	22	50	9
**Case 20**	22	54	6
**Case 21**	20	51	9
**Case 22**	22	51	9

Supplementary data Table S2: Effect of mutations on trans-membrane topology of mutated proteins

Case #	No. of TM segments	Length of signal peptide	No. of amino acids in TB segments		
			S1	S2	S3
Normal	2	1-33	205-220	244-263	---
1	---	---	---	---	---
2	2	1-38	207-222	246-266	---
3	---	---	---	---	---
4	---	---	---	---	---
5	---	---	---	---	---
6	---	---	---	---	---
7	1	1-29	202-221	---	---
8	---	---	---	---	---
9	3	1-33	205-220	244-262	346-361
10	1	1-28	206-221	---	---
11	2	1-33	206-221	244-259	---
12	2	1-33	206-221	244-259	---

13	2	1-33	205-220	243-259	---
14	2	1-36	205-220	244-260	---
15	2	1-37	205-220	244-261	---
16	2	1-37	205--220	244-263	---
17	2	1-37	205--220	244-263	---
18	2	1-33	205-220	244-264	---
19	2	1-33	205-220	244-264	---
20	2	1-33	205-220	244-264	---
21	2	1-33	205-220	244-264	---
22	2	1-37	205--220	244-263	---

TM = transmembrane
